# An Ingenious Super Light Trapping Surface Templated from Butterfly Wing Scales

**DOI:** 10.1186/s11671-015-1052-7

**Published:** 2015-08-26

**Authors:** Zhiwu Han, Bo Li, Zhengzhi Mu, Meng Yang, Shichao Niu, Junqiu Zhang, Luquan Ren

**Affiliations:** Key Laboratory of Bionic Engineering (Ministry of Education, China), Jilin University, Changchun, 130022 P. R. China

**Keywords:** Bio-template fabrication, Butterfly wing scales, Light trapping materials

## Abstract

Based on the super light trapping property of butterfly *Trogonoptera brookiana* wings, the SiO_2_ replica of this bionic functional surface was successfully synthesized using a simple and highly effective synthesis method combining a sol–gel process and subsequent selective etching. Firstly, the reflectivity of butterfly wing scales was carefully examined. It was found that the whole reflectance spectroscopy of the butterfly wings showed a lower level (less than 10 %) in the visible spectrum. Thus, it was confirmed that the butterfly wings possessed a super light trapping effect. Afterwards, the morphologies and detailed architectures of the butterfly wing scales were carefully investigated using the ultra-depth three-dimensional (3D) microscope and field emission scanning electronic microscopy (FESEM). It was composed by the parallel ridges and quasi-honeycomb-like structure between them. Based on the biological properties and function above, an exact SiO_2_ negative replica was fabricated through a synthesis method combining a sol–gel process and subsequent selective etching. At last, the comparative analysis of morphology feature size and the reflectance spectroscopy between the SiO_2_ negative replica and the flat plate was conducted. It could be concluded that the SiO_2_ negative replica inherited not only the original super light trapping architectures, but also the super light trapping characteristics of bio-template. This work may open up an avenue for the design and fabrication of super light trapping materials and encourage people to look for more super light trapping architectures in nature.

## Background

Nature offers a variety of surfaces with brilliant properties and is a source of inspiration for numerous applications and techniques. A number of researchers have paid close attention to the fantastic surfaces triggered by nature, such as the anisotropy of the rice leaves [1, 2], the self-cleaning of the lotus leaves [3–5], the antireflection of moth eyes [6–8], the fog collection system in cactus [9, 10], the reversible adhesive of the gecko feet [11, 12], the superhydrophobicity of the water strider legs [13, 14], and the iridescence of the butterfly wings [15–17]. Mimicking or studying the basic principles of the sophisticated tactics from nature is of significant importance for the design of artificial analogs.

During the last few decades, much effort has been directed toward the brightest and most vivid structure-based colors in nature that arise from the interaction of light with surfaces on the micro- and nanoscale. The architectures on the surface of butterfly wings not only formed the gorgeous appearance, but also made the butterfly wings with appealing properties, such as observable optical response to temperature [18], highly selective vapor response [19, 20], and light trapping effect [21, 22]. Although many scientists have done a lot of research on the architectures of butterfly wings and their multi-functional features, little attention has been paid to the black color in spite of its ubiquity. Many scientists have ascribed the blackness of butterfly wings to melanin solely, until recent research results indicated that the nanostructures of scales also play a key role in the exhibition of blackness [23–25]. In addition, seeking for light trapping surfaces has been a great challenge for researchers and now the research on light trapping materials has attracted more and more people’s attention because of the increasing importance of light trapping materials. Considering the above, we chose butterfly *Trogonoptera brookiana* black wings as a natural model and revealed its properties for super light trapping.

On the other hand, although the color mechanism and structural characterizations have been well investigated for a long time [26], the studies on bionic preparation of the subtle nanostructures on butterfly wing scales have been greatly restricted. In fact, the exact combination of the three-dimensional (3D) structure and cuticle complex refractive index [n* = (1.55 ± 0.05) + *i* (0.06 ± 0.01)] is beyond the capabilities of existing nanofabrication techniques [27]. In spite of this, the potential valuable application prospect still inspired scientists to devote themselves to mimicking the distinctive surface nanostructure of butterfly wings. The colorful butterfly wing surface has been fabricated using soft lithography technique [28], low-temperature atomic layer deposition [29], colloidal self-assembly, sputtering and atomic layer deposition, even combining all these layer deposition techniques together [30]. Not only sorts of surface processing technology but also a variety of materials were employed to fabricate replicas of the multi-layered scales on the surface of butterfly wing, such as polyelectrolyte multilayer films [31], carbon nanotube [32], polypyrrole [33], oxidizing material include TiO_2_ [34], Bi_2_WO_6_ [35], Fe_3_O_4_ [36], SnO_2_ [19], and so on. Even so, exact replica of such biological structures by an artificial synthesis route are difficult; what is more, the existing studies about butterflies mainly focus on the structural colors, photonic crystal structures, and the replica of photonic structures in butterfly wings. Few researchers showed solicitude for the bionic fabricating of the super light trapping architectures in butterfly wings. So, it is urgently necessary to develop a high-efficiency and low-cost technique to realize the outstanding light trapping architectures.

In this work, we selected the butterfly *Trogonoptera brookiana* black wings as the bio-templates, and the super light trapping characteristics were characterized by reflectance spectroscopy. Besides, the super light trapping architectures and morphology of the wing scales were characterized by FESEM. In order to prepare the SiO_2_ negative replica, a simple and highly effective synthesis method combining a sol–gel process and subsequent selective etching was adopted. What is more, the 3D optimized models were generated by CATIA to illustrate the super light trapping architectures and fabrication process effectively. At last, the reflectance spectroscopy of SiO_2_ negative replica and a flat plate was measured. The SiO_2_ negative replica was not only stable but also corrosion resistant due to the complex super light trapping architectures and inert SiO_2_ material, which made it a promising super light trapping surface for various fields such as photoelectrical devices, photo-induced sensors and solar cells. Moreover, this work sets up a strategy for the design and fabrication of super light trapping materials and may encourage people to look for more super light trapping architectures in nature.

## Methods

### Materials

Analytic grade reagents, hydrochloric acid, and tetraethyl ortho-silicate were provided by Beijing Chemical Works. Ethanol absolute, diethyl ether, concentrated nitric acid, and perchloric acid were provided by Tianjin Fine Chemical Co., Ltd.

Although the green wings of butterfly *Trogonoptera brookiana* possess a light trapping property which was confirmed as reported in our previous work [22], this study found that the black wing scales had the better light trapping characteristic compared with the previous study. Thus, the black wings were selected as a biological prototype in this work. Butterfly wing samples of uniform size (15 mm in length and 10 mm in width, rectangular) were cut off from the black areas in a perpendicular and parallel direction to the ridge veins, respectively. In order to confirm that the butterfly wing samples were clean, some pre-processing was conducted. Firstly, each sample was soaked by aether for 10 min to remove proteins and fattiness on the samples’ surface. Afterwards, the dehydration treatment in ethanol absolute with duration time of 15 min for each specimen was taken. The purpose of conducting the dehydration pre-processing was to increase the mechanical strength and stability of the treated tissues. Then, the samples were dried naturally in air.

### Discoloration Experiment

A simple discoloration experiment was carried out to confirm that the color of the black wing scales was structure-based rather than pigment. Firstly, the neat and clean black areas were cut off from the butterfly wings meticulously with a scalpel in perpendicular and parallel directions to the nervure, respectively. Then, the sample was clamped with a tweezer to flatwise place in a petri dish and soaked in a certain amount of diethyl ether and ethanol absolute for 10 min, respectively, for degreasing and increasing the mechanical strength of the wing tissues. The color of the air-dried sample was still black, which was not affected by organic solvents virtually.

### Preparation of the SiO_2_ Negative Replica

Firstly, the butterfly wing samples were sandwiched between two glass slides of which both ends were clamped by clips with proper force. Using a micropipette, a suitable amount of the sol–gel precursor solution, a reaction product of tetraethyl ortho-silicate and hydrochloric acid (3:1 in volume), was added to the edge of the assembly with a volume of 4–8 μL. Then, the assembly was heated at 125 °C for 25 min in an electric vacuum-drying oven to further solidify the precursor solution on the surface of the wing samples. Next, the whole assembly was dipped into a mixture of concentrated nitric acid and perchloric acid (1:1 in volume) while heating at 130 °C for 40 min to remove the original organisms. Then, the whole assembly was washed by ultrasonic oscillation for 5 min in deionized water to get rid of the residue. After drying at room temperature, the SiO_2_ negative replica was fabricated.

### Spectroscopy Characterization

The reflectance spectroscopy of butterfly *Trogonoptera brookiana* wings and the SiO_2_ negative replica were measured using a miniature fiber optic spectrometer (Ocean Optics USB4000-VIS-NIR) equipped with a halogen tungsten lamp source (Ocean Optics LS-1-LL). The spot size of the incident beam was ~2 mm, and the wavelength of the reflectance spectroscopy varied in the range of 400–900 nm. In addition, the element types and content analysis of the surface of the SiO_2_ negative replica were characterized by virtue of the energy dispersive spectroscopy (EDS, OXFORD X-Max^N^ 150) on the SEM.

### Morphology and Dimension Characterization

The 2D morphologies and structures of the scales of the black areas of butterfly *Trogonoptera brookiana* hind wings and the SiO_2_ negative replica were obtained with the help of field emission scanning electronic microscopy (FESEM: JEOL JSM-6700 F). These data will be used to analyze the inheritance accuracy of the SiO_2_ negative replica.

## Results and Discussion

### Macroscopic Morphology Observations and Reflectance Spectroscopy Analysis of Black Butterfly Wing Scales

**Fig. 1:**
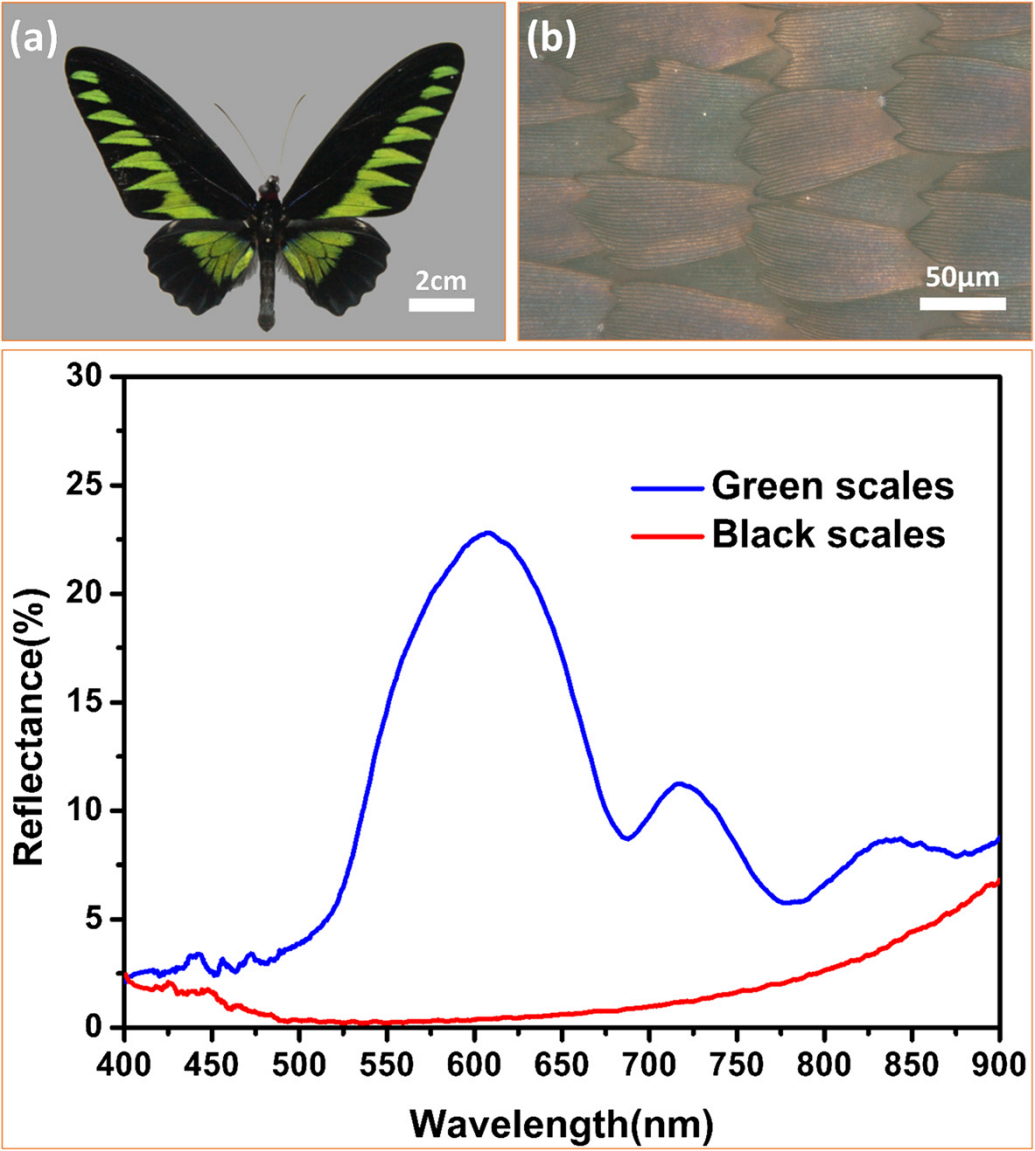
The macroscopic morphology of the butterfly wings and the reflectance spectroscopy of black and green region of the butterfly wings. **a** Photograph of butterfly *Trogonoptera brookiana*. **b** Optical microscopic image of the black butterfly wing scales. **c** The lower reflectance of the black wing scales was confirmed in the entire wave range

Figure 1a showed the overall view of the original butterfly *Trogonoptera brookiana*. Obviously, there were long smooth black strips located at the front and hind wings of the butterfly, which looked as beautiful as black velvet. With the help of optical metallurgical microscopy, it could be found that the black part of the wings was covered by bright black scales. These black scales were placed in alternate rows, and the scales overlap each other, as shown in Fig. 1b. The reflectance spectroscopy ranging from 400–900 nm of the butterfly wings is shown in Fig. 1c. It was found that the reflectivity of black wings was lower than that of green wings, which was less than 8 % in the range of 400–900 nm. Based upon the butterfly being a kind of poikilothermal animal, it could be inferred that lower reflectance made contributions to reducing the loss of solar energy so that the butterfly could maintain body temperature, which confirmed that the black areas possess a better light trapping property. Hence, the black areas were chosen as the experimental areas to be studied carefully.

### Microscopic Morphology Observations of Black Butterfly Wing Scales

**Fig. 2:**
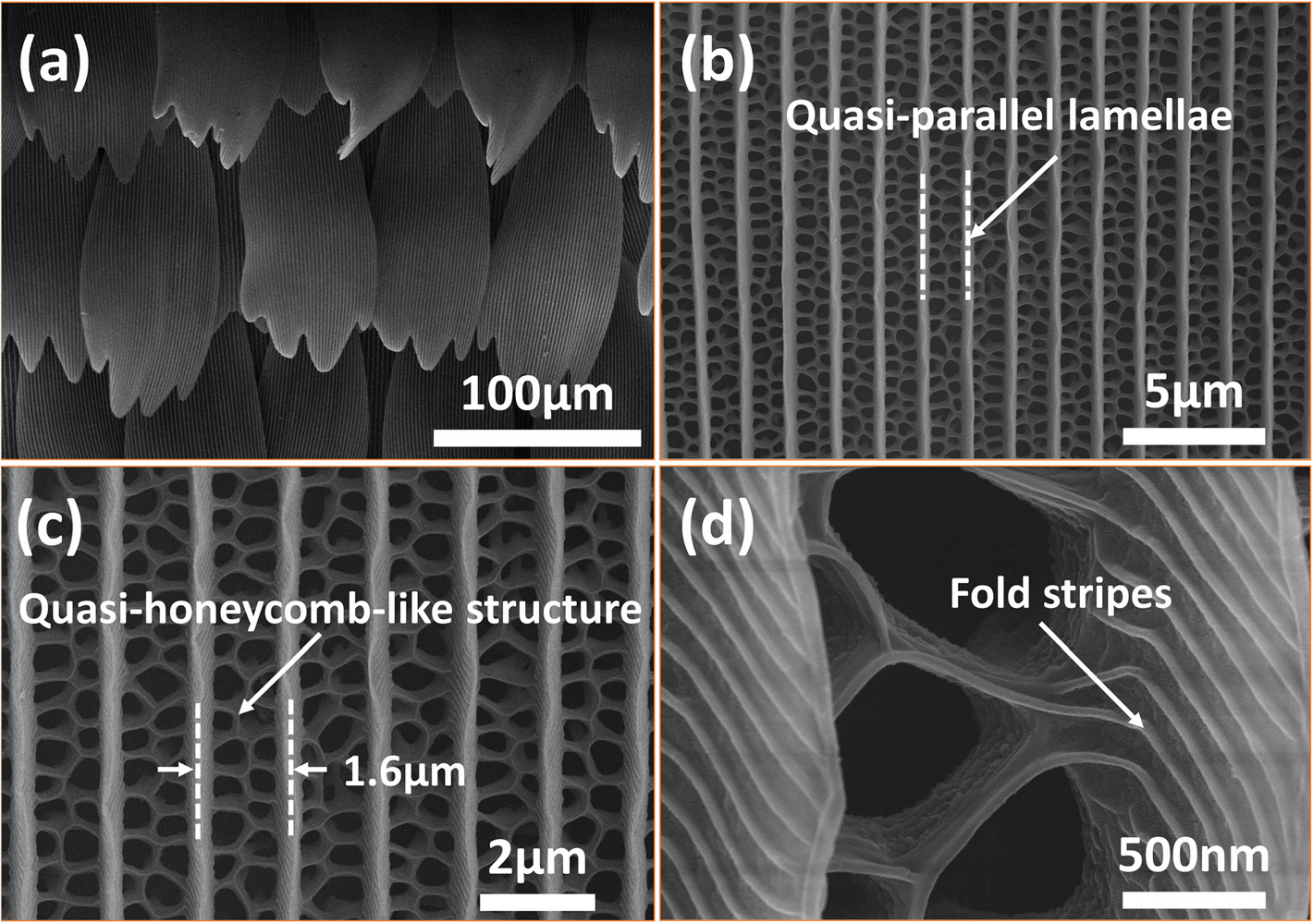
FESEM images of original butterfly scales in the black region with different magnifications. **a** Lower magnification image. It was found clearly that these scales ranged in good order on the substrate. **b**, **c** Medium magnification image. This surface of the scale comprised a set of raised longitudinal quasi-parallel lamellae (ridges), the space between which was filled with a quasi-honeycomb-like structure. **d** The high-magnification images of the quasi-honeycomb-like structure

In order to obtain more detailed information about the black scales’ nanostructures, FESEM was employed for the characterization of the morphology and distribution of the black scales, as shown in Fig. 2. It was clearly found that these scales ranged in good order on the substrate under low magnification. The scales with an area of about 160 μm in length and 70 μm in width had sharp serrate ends. The root of each scale was embedded on the substrate, as shown in Fig. 2a. More exquisite nanostructures of the black scales were observed under medium magnification as shown in Fig. 2b, c. It could be observed that the scales had longitudinal ridges, which run through the scales. The distance between two adjacent ridges was approximately 1.6 μm. This surface of the scale comprised a set of raised longitudinal quasi-parallel lamellae (ridges). The space between adjacent ridges was filled with a netlike reticulum composed of pores. As discussed further in this paper, the reticulum and lamellae were both the optical elements that endowed the wing scales a super light trapping effect. It was called a quasi-honeycomb-like structure. Figure 2d shows the high-magnification images of the quasi-honeycomb-like structure. It could be observed clearly that there were parallel fold stripes on both sides of the ridges. These fold stripes could be the proof if whether the original nanostructures in bio-templates were faithfully inherited by the replica or not.

### The Formation Mechanism of the SiO_2_ Negative Replica

**Fig. 3:**
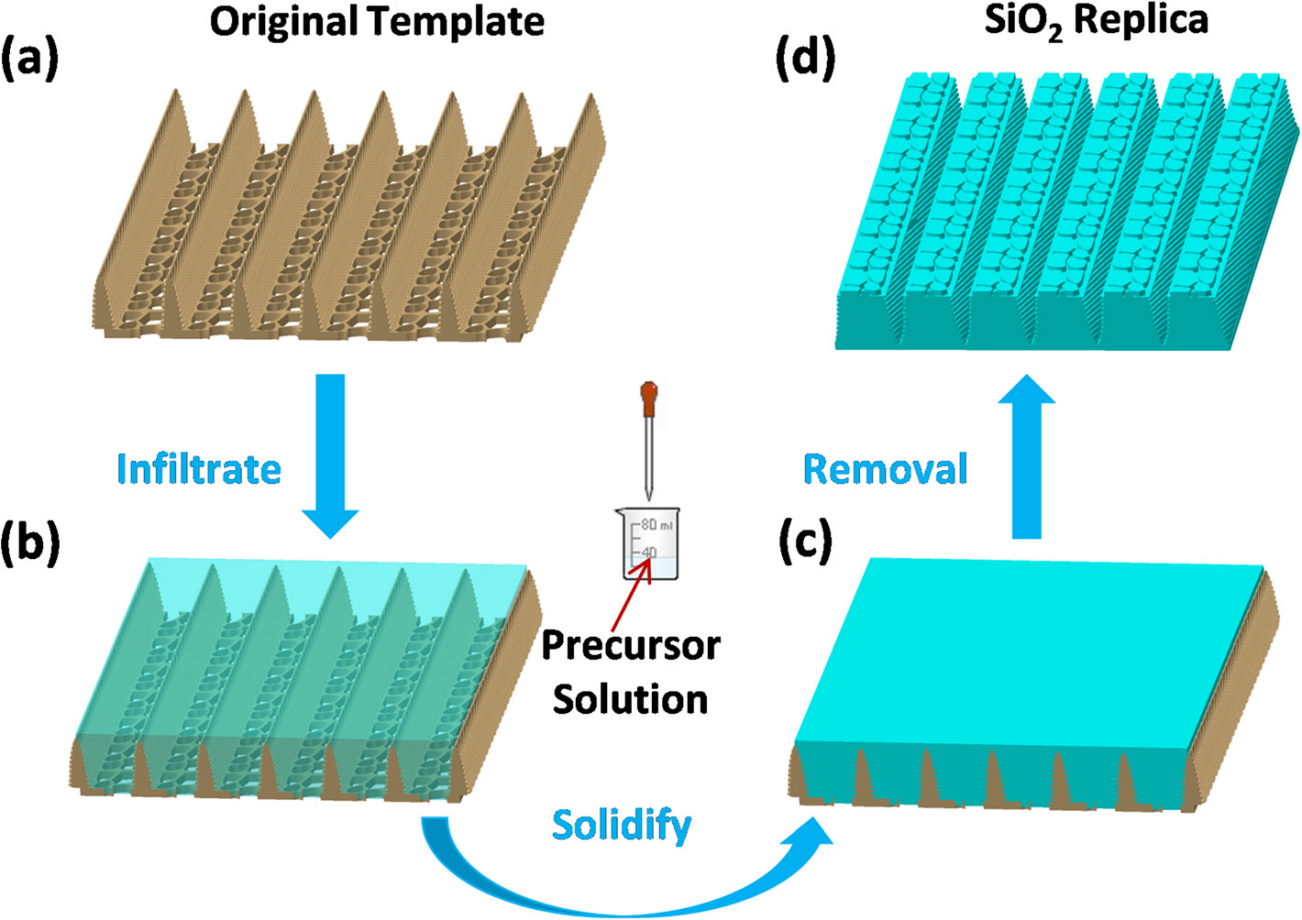
Fabrication process from the original templates of butterfly wings to the SiO_2_ negative replica. **a** 3D nanostructured model of the original butterfly wings. **b** The precursor solution filled the space left between the micro-ridges with a micropipettor. **c** The precursor solution became solidified through heating process. **d** The solid negative replica was obtained after the original bio-templates were etched away and cooled at room temperature

As shown in Fig. 3, 3D CATIA models were built according to the overall process of the preparation using bio-templates from butterfly *Trogonoptera brookiana* wings: (a) the clean original templates (3 cm × 2 cm), which was pretreated with diethyl ether and ethanol absolute for 10 min, respectively, was sandwiched between two glass slides; (b) a suitable amount of the precursor solution (~8 μL) was added to the edge of the assembly using a micropipette; (c) the assembly was heated at 125 °C for 25 min in an electric vacuum-drying oven to solidify the precursor solution; (d) the original template was removed through the process of selective etching in a mixture of concentrated nitric acid and perchloric acid (1:1 in volume) at 130 °C for another 40 min. Then, the whole assembly was washed by ultrasonic oscillation for 5 min in deionized water to get rid of the residue. Finally, the whole assembly was washed with deionized water for 15 min. After drying at room temperature, the SiO_2_ negative replica was fabricated.

### Microscopic Morphology Observations and EDS Analysis of the SiO_2_ Negative Replica

**Fig. 4:**
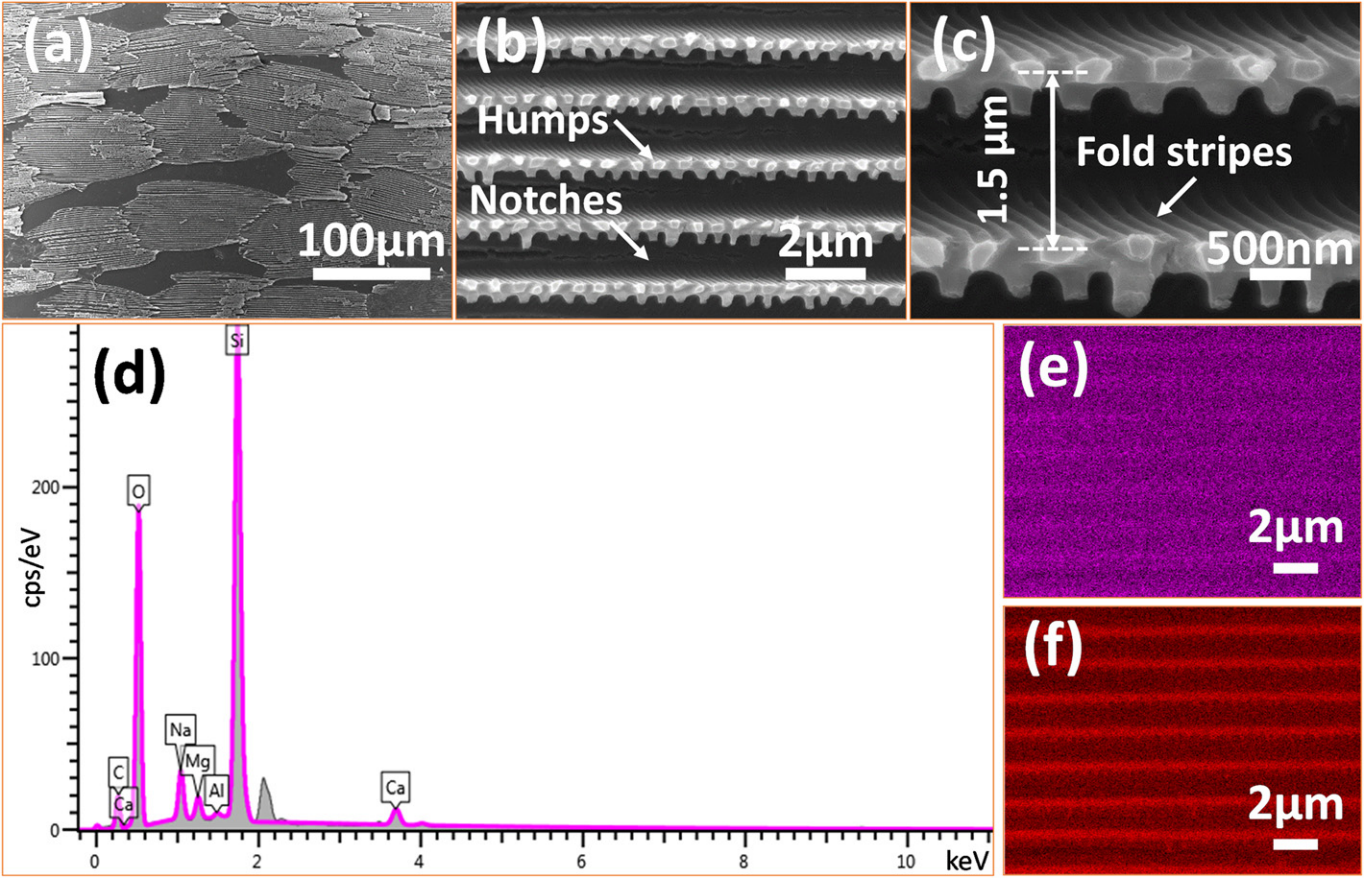
FESEM images and EDS spectrum of the SiO_2_ negative replica. **a** Lower magnification image. It could be found that the scales were still arranged in rows. However, they were no longer overlapped with each other. **b** Medium magnification image of the SiO_2_ negative replica. It can be observed that notches are lying in parallel, with humps of different shapes between them. **c** High-magnification images of the replica surface. The sizes and shapes of the humps were in conformity with those of the pores shown in Fig. 2c. **d** The EDS spectrum showed the main elements constituting the SiO_2_ negative replicas. **e, f** The scanning maps of silicon and oxygenium demonstrated the distribution of silicon and oxygenium, which was consistent with the structures shape of the subwavelength antireflective nanoditches arrays

The morphology of the SiO_2_ negative replica was investigated by FESEM, and the result is illustrated in Fig. 4a. Some scale-like structures were distributed on the surface of the replica, the shapes of which were similar to those of the black scales shown in Fig. 1b. From the aspect of the arrangement, the scaly structures of the replica also had good periodicity and arranged regularly from the front to the end of the replica in the same sequence just like tiles on the roof, which was also analogous with the bio-templates. The structural details of the SiO_2_ negative replica of the single scale were illustrated in Fig. 4b under medium magnification. It could be observed that notches were lying in parallel, with humps of different shapes between them. These notches were formed from the ridges on the scales of the black wings. The sol–gel precursor filled the space left between the ridges and became solidified, making the places that used to be ridges became notches, and the pores between ridges became humps between notches. Figure 4c is a high-magnification image of the negative replica of the black wing scales. The period of the negative replica nanostructures was about 1.5 μm. The size and shape of the humps were in conformity with those of the pores shown in Fig. 2c, which confirmed that these humps were the reverse structures of those pores. It was worth to mention that parallel nanostructures were obtained on both sides of the notches. These nanostructures were formed from fold stripes on both sides of the ridges mentioned when illustrating the morphology of black wing scales (Fig. 2d). In a word, after comparing Fig. 4 with Fig. 2 from a variety of angles, such as appearance, arrangement, size of scales, notches, and bumps, it was obvious that the original super light trapping architectures of bio-template were well inherited by the SiO_2_ negative replica. What is more, the appearance and comparison of the fold stripes on both sides of the ridges also draw the conclusion that the original architectures in bio-template were faithfully inherited by the SiO_2_ negative replica. To the best of our knowledge, this is the first time that a SiO_2_ negative replica of the original black butterfly *Trogonoptera brookiana* wing scales has been synthesized. However, the size of the negative replica was a bit different from the original scales. The element types and content analysis of the surface of the SiO_2_ negative replica were characterized with the help of an energy dispersive spectrometer (EDS). The EDS microanalyses (Fig. 4d) of the SiO_2_ replica indicated that the SiO_2_ replica was mainly composed of silicon and oxygenium. Peaks of silicon and oxygenium could be observed clearly, and the weight percentages of these two elements were 31.32 and 45.31 %, respectively. And the element enrichment regions of both silicon and oxygenium were consistent with the shape of the SiO_2_ negative replicas as the area scanning maps shown in Fig. 4e, f, which further indicated that highly purified SiO_2_ replicas were obtained.

### Analysis of the Light Trapping Mechanism of the SiO_2_ Negative Replicas

**Fig. 5:**
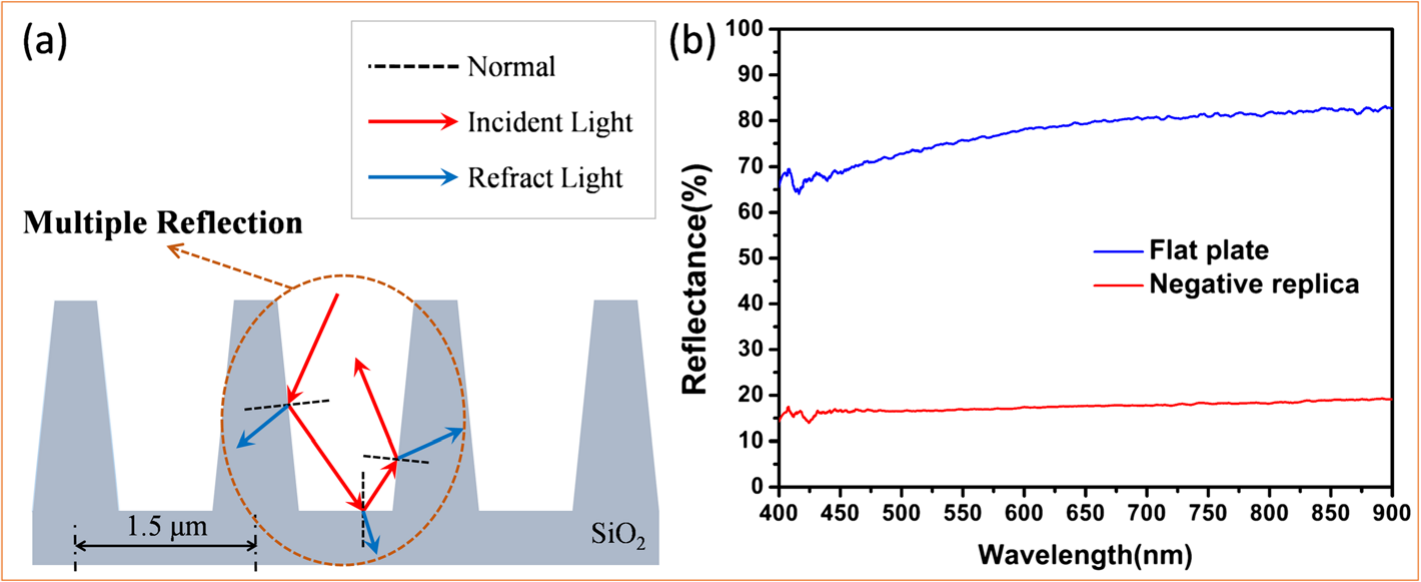
Schematic illustration of the multiple reflection and refraction occurred in the SiO_2_ negative replicas and the reflectance spectroscopy analysis of the SiO_2_ negative replica. **a** After multiple reflections and refractions, incident light traveled for a longer distance, and only a small part of the solar energy was reflected back to the air. **b** The average reflection of the SiO_2_ negative replicas was about 20 % which was just 1/4 of the reflection of the flat plate without the negative quasi-honeycomb-like structure

The simplified model of the SiO_2_ negative replicas was built as shown in Fig. 5a. When incident light traveled through air to the SiO_2_ material, reflection and refraction would happen on the interface at the same time. After multiple reflections and refractions, incident light traveled for a longer distance. Only a small part of the solar energy was reflected back to the air, resulting in most of the incident light being effectively adsorbed within the super light trapping architectures eventually. What is more, the humps on the top of the ridges and the fold stripes on both sides of the ridges enhanced the scattering of incident light within super light trapping architectures of the SiO_2_ negative replicas, which also reduced the solar energy loss (SEL) due to the reflectance from the SiO_2_ negative replica.

Measured reflectance spectroscopy in Fig. 5b shows that the applying of the negative quasi-honeycomb-like structure on flat plate played the key role in producing the super light trapping property. The red curve shows that the average reflection of the SiO_2_ negative replica was about 20 %, and the blue curve shows that the average reflection of the flat plate was about 80 %. It was obtained that the average reflection of the SiO_2_ negative replica was just 1/4 of the reflection of the flat plate without the negative quasi-honeycomb-like structure. To further clarify the influence of the negative quasi-honeycomb-like structure on the super light trapping property of the SiO_2_ negative replica, the SEL caused by the reflection of the SiO_2_ negative replica and the flat plate was calculated according to the equation below, where *I* (*λ*) is the solar energy intensity as a function of the wavelength *λ* at AM1.5 and *α* (*λ*) is the measured reflectance of the SiO_2_ negative replica, and the flat plate is a function of the wavelength *λ* [37–40].

$$ SEL={\displaystyle \underset{400}{\overset{900}{\int }}I\left(\lambda \right)\alpha \left(\lambda \right)d\lambda } $$

The SEL calculated from the measured reflectance spectroscopy (seen in Fig. 5b) of the flat plate and the SiO_2_ negative replica were 491.34 and 110.98 W/m^2^, respectively. Apparently, the SEL of the flat plate was more than four times the SEL of the SiO_2_ negative replica, which confirmed that the SiO_2_ negative replica had the better light trapping characteristics. Given that they shared uniform intensity of the light and material, it could be inferred from the results that the negative quasi-honeycomb-like structure which was borrowed from the black wing scales of butterfly acted as super light trapping architectures. Thus, it could be concluded that the SiO_2_ negative replica inherited not only the original super light trapping architectures, but also the super light trapping characteristics of bio-template.

## Conclusions

In summary, the reflectivity of the black wings of butterfly *Trogonoptera brookiana* was carefully examined, and the wings showed good optical absorption in the visible spectrum, which confirmed that the black wings of this butterfly possessed structure-based super light trapping property. So, a super light trapping material was then fabricated templated from these black wings by transferring the quasi-honeycomb-like structure from the black wing scales to flat plate. The structural replication fidelity of the process is demonstrated on both the macro- and microscale, and even down to the nanoscale, as evidenced by FESEM and energy dispersive spectroscopy (EDS). The comparison results of feature size between the SiO_2_ negative replica and original bio-templates were in concordance to some extent. Considering of the final observation result, the obtained SiO_2_ negative replica preserved the super light trapping architectures successfully from the aspects of morphology, dimensions, and distributions of the scales. At last, the reflectance spectroscopy of the SiO_2_ negative replica and a flat plate was measured. It was found that the SiO_2_ negative replica obtained 20 % reflection in visible light spectrum. Reflection of the SiO_2_ negative replica with the negative quasi-honeycomb-like structure was merely 1/4 of that in the flat plate. Thus, it was obvious that the obtained SiO_2_ negative replica possessed super light trapping property. From the analysis above, it was proved that the super light trapping surface of original bio-templates was also inherited by the SiO_2_ negative replica faithfully in terms of structure and function. Thus, it could be concluded that the SiO_2_ negative replica inherited not only the original super light trapping architectures, but also the super light trapping characteristics of bio-template.

The manufacture of the super light trapping architectures of butterfly *Trogonoptera brookiana* black wing scales is meaningful. The SiO_2_ negative replica was not only stable but also corrosion resistant due to the complex super light trapping architectures and inert SiO_2_ material, which made it a promising light trapping surface for various fields such as photoelectrical devices, photo-induced sensors, and solar cells. Moreover, this work sets up a strategy for the design and fabrication of super light trapping materials and may encourage people to look for more super light trapping architectures in nature.

## Abbreviations

SEM: scanning electron microscope
FESEM: field emission scanning electron microscope
EDS: energy dispersive spectroscopy
ALD: atomic layer deposition
